# The aqueous stability and interactions of organoruthenium compounds with serum proteins, cell culture medium, and human serum

**DOI:** 10.1093/mtomcs/mfac043

**Published:** 2022-07-25

**Authors:** Mie Riisom, Liam Eade, William D J Tremlett, Christian G Hartinger

**Affiliations:** School of Chemical Sciences, University of Auckland, 23 Symonds Street, Auckland 1010, New Zealand; School of Chemical Sciences, University of Auckland, 23 Symonds Street, Auckland 1010, New Zealand; School of Chemical Sciences, University of Auckland, 23 Symonds Street, Auckland 1010, New Zealand; School of Chemical Sciences, University of Auckland, 23 Symonds Street, Auckland 1010, New Zealand

**Keywords:** anticancer compounds, capillary electrophoresis, mass spectrometry, metallomics, organoruthenium complexes, serum proteins

## Abstract

Metal complexes bind to a wide variety of biomolecules and the control of the reactivity is essential when designing anticancer metallodrugs with a specific mode of action in mind. In this study, we used the highly cytotoxic compound [Ru^II^(cym)(8-HQ)Cl] (cym = η^6^-*p*-cymene, 8-HQ = 8-hydroxyquinoline), the more inert derivative [Ru^II^(cym)(8-HQ)(PTA)](SO_3_CF_3_) (PTA = 1,3,5-triaza-7-phosphaadamantane), and [Ru^II^(cym)(PCA)Cl]Cl (PCA = pyridinecarbothioamide) as a complex with a different coordination environment about the Ru center and investigated their stability, interactions with proteins, and behavior in medium (αMEM) and human serum by capillary zone electrophoresis. The developed method was found to be robust and provides a quick and low-cost technique to monitor the interactions of such complexes with biomolecules. Each complex was found to behave very differently, emphasizing the importance of the choice of ligands and demonstrating the applicability of the developed method. Additionally, the human serum albumin binding site preference of [Ru^II^(cym)(8-HQ)Cl] was investigated through displacement studies, revealing that the compound was able to bind to both sites I and site II, and the type of adducts formed with transferrin was determined by mass spectrometry.

## Graphical Abstract

**Graphical Abstract:**
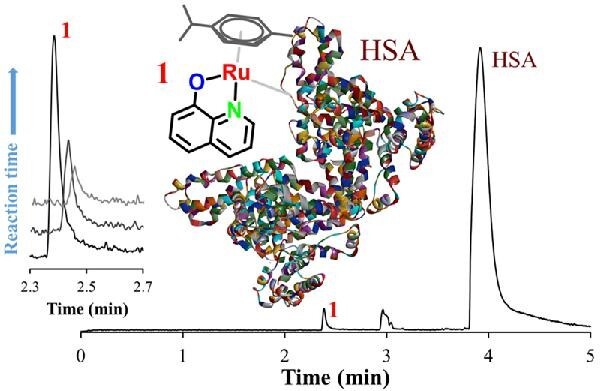
Tracking the binding of [Ru(cym)(8-HQ)Cl] to human serum albumin over time using capillary electrophoresis.

## Introduction

**Fig. 1:**
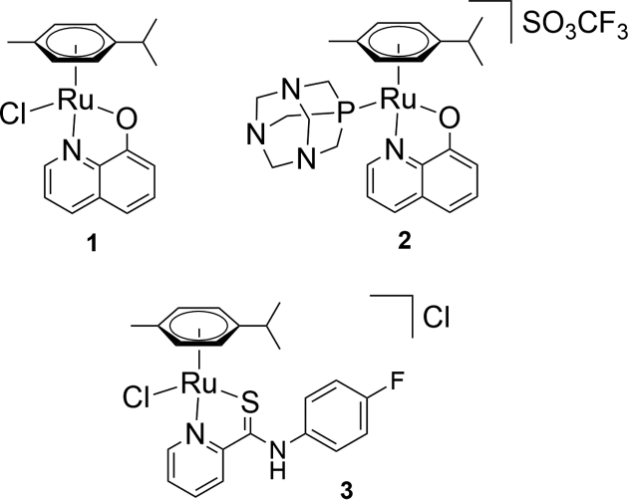
Chemical structures of [Ru^II^(cym)(8-HQ)Cl] **1**, [Ru^II^(cym)(8-HQ)(PTA)](SO_3_CF_3_) **2**, and [Ru^II^(cym)(PCA)Cl]Cl **3**.

Pt-based anticancer drugs are among the most widely used chemotherapeutics.^1^^,^^2^ Although cisplatin, oxaliplatin, and carboplatin are very potent, they are also associated with severe side effects and acquired or intrinsic resistance. Over recent decades, there has been a high interest in developing Ru-based anticancer complexes, as they offer novel mechanisms of action and reduced toxicity,^3–5^ and some Ru complexes have shown efficacy in tumors resistant to treatment with Pt-based drugs.^6^ Ru piano-stool organometallics based on the [Ru(cym)Cl] moiety (cym = η^6^-*p*-cymene) of bioactive 8-oxyquinoline (8-HQ)-derived ligands or with a *N*-substituted 2-pyridinecarbothioamide (PCA) ligand have recently attracted attention as potential new anticancer agents.^7^ We have reported the biological activity of several derivatives including [Ru^II^(cym)(8-HQ)Cl] **1** and its more water-soluble PTA derivative [Ru^II^(cym)(8-HQ)(PTA)](SO_3_CF_3_) **2** (PTA = 1,3,5-triaza-7-phosphaadamantane), as well as [Ru^II^(cym)(PCA)Cl]Cl **3** (Fig. 1).^8–11^ Complexes **1** and **3** have shown promising antiproliferative properties with IC_50_ values in the low micromolar range,^7^^,^^9^ whereas complex **2** is less cytotoxic, which can be attributed to its lower lipophilicity and thereby lower ability to cross the cell membrane.^10^ Furthermore, complex **3** has shown potential as a tumor invasiveness inhibitor as it has been confirmed to specifically target the scaffold protein and cytolinker plectin.^8^

Overall, the mechanisms of action of organoruthenium compounds are still not entirely understood, and additional studies investigating the interactions of these complexes in biological environments are therefore highly relevant. Many chemotherapeutic drugs are developed for intravenous administration and it is therefore important to study their interactions with some of the first potential binding partners the drugs will encounter when entering the blood stream, in particular the abundant human serum proteins albumin (HSA) and transferrin (Tf).^12^ For Pt anticancer compounds, the binding to proteins such as albumin has largely been interpreted as an inactivation of the drug by reducing the bioavailability.^13^^,^^14^ For Ru complexes, it has been questioned whether binding to proteins can actually enhance the cellular uptake of the drug through specific transporters or if protein binding might act as a reservoir, which could allow the complex to remain in the bloodstream for longer.^6^^,^^15^ Several studies have investigated whether the binding of a Ru anticancer complex to Tf is an advantage for targeting tumor cells, as rapidly growing cells have a higher demand for iron and display more Tf receptors, but have so far been unable to verify that Tf plays a role in the activity of these complexes.^6^^,^^16^^,^^17^ Albumin, which is the most abundant serum protein, transports several different biomolecules such as fatty acids, amino acids, steroids, and metals, and additionally has the ability to transport many pharmaceuticals.^18^^,^^19^ Albumin consists of three similar domains (I, II, and III), with each domain having two subdomains (denoted a or b).^18^^,^^19^ Ligand binding often occurs at the hydrophobic cavities in subdomains IIa (site I) or IIIa (site II). By using well-established binding site markers for albumin, such as warfarin (Wf; site I)^18^ and dansylglycine (Ds; site II),^20^ displacement studies provide information on preferred binding sites.^21^

Capillary electrophoresis (CE) is a very attractive technique for studying the speciation of metallodrugs in biological media, as it is a powerful separation technique and compatible with aqueous conditions, which makes it possible to monitor reactions under simulated physiological conditions.^22^ CE and CE hyphenated to mass spectrometry (MS) have been used to investigate the binding of metal-based anticancer agents to different biological targets such as DNA or proteins such as albumin, ubiquitin, and Tf .^21^^,^^23–26^

In this study, a CE method applicable for investigating the interactions of anticancer organoruthenium compounds with biomolecules was developed and validated. The developed method provides a quick and low-cost technique to monitor the interactions of new potential metal-based anticancer complexes with biomolecules, which can be a first step toward understanding their mechanisms of action. Additionally, the HSA binding site preference of [Ru^II^(cym)(8-HQ)Cl] **1** was investigated through displacement studies and the types of adducts formed with Tf were determined by MS.

## Materials and methods

### Reagents and materials

[Ru^II^(cym)(8-HQ)Cl] **1**, [Ru^II^(cym)(8-HQ)(PTA)](SO_3_CF_3_) **2**, [Ru^II^(cym)(PCA)Cl]Cl **3**, and the internal standard [tris(acetylacetonato)cobalt(III)] [Co(acac)_3_] were prepared according to literature procedures.^8–10^^,^^27^ Water (18 MΩ) used throughout these experiments was obtained from a Millipore Milli-Q Gradient Water Purification System. Methanol was purchased from Macron Fine Chemicals^TM^. The background electrolyte (BGE) for the CE studies was a 20 mM phosphate buffer (pH 7.4) which was prepared from H_2_O (18 MΩ), NaH_2_PO_4_·2H_2_O (99%, AK Scientific), and Na_2_HPO_4_·2H_2_O (≥99.5%, Sigma-Aldrich). Reagents used for CE conditioning were NaOH (1.0 M, Agilent Technologies) and HCl (36.5-38%, J.T. Baker).

Protein interaction studies were carried out using HSA (≥99%, Sigma-Aldrich, A3782 fatty acid free) and Tf (≥98%, Sigma-Aldrich). Interactions with cell medium and human serum were investigated using αMEM cell culture medium (Life Technologies) spiked with 5% fetal bovine serum (FBS) (Moregate BioTech), and human serum (from human male AB plasma, US origin, sterile filtered, Sigma-Aldrich), respectively. HSA binding site displacement studies were conducted with Wf (≥97%, Sigma-Aldrich), Ds (≥99.5%, Sigma-Aldrich), and HSA (lyophilized powder, ≥96%, Sigma-Aldrich, A1653).

### Stability studies

Stability studies of complexes **1**–**3** were carried out in both H_2_O and 20 mM phosphate buffer (pH 7.4). Stock solutions for the complexes were prepared in methanol (2 mM). The samples were diluted just before the first measurement to a final complex concentration of 200 μM. Tris(acetylacetonato)cobalt(III) [Co(acac)_3_] was added as an internal standard to all samples at a final concentration of 100 μM. The stability was studied for 24 h by monitoring changes in the peak areas over time.

### Interactions with serum proteins, cell culture medium, and human serum

Stock solutions of HSA and Tf in BGE were prepared (400 μM). Studies were performed for **1** and **2** at complex (200 μM):protein ratios of 1:1, 10:1, and 20:1. The PCA complex **3** was investigated at complex (200 μM):protein ratios of 20:1 and 50:1 for HSA and at 10:1 and 20:1 for Tf. Samples were prepared just before the first measurement. BGE was used for dilution and [Co(acac)_3_] was added as the internal standard to all samples at a final concentration of 100 μM.

The interactions of complexes **1**–**3** with cell culture medium (αMEM with 5% FBS) were investigated. The αMEM was diluted 1:10 with BGE and the complex concentrations were kept at 200 μM and [Co(acac)_3_] at 100 μM. The dilution corresponded to a complex:albumin ratio of 133:1.

The interactions of complexes **1**–**3** with human serum were investigated after diluting the serum 1:100 with BGE and the complex concentrations were kept at 200 μM and for [Co(acac)_3_] at 100 μM. The dilution corresponded to a complex:protein ratio of about 50:1 with regard to albumin content.

### Competitive displacement study for HSA binding sites

The affinity of complex **1** for binding sites I and II on HSA was investigated with Wf as the site marker for binding site I and Ds for site II by tracking the peak area for unbound Wf and Ds, respectively. Stock solutions of Wf, Ds, and HSA were prepared in H_2_O at concentrations of 800, 800, and 200 μM, respectively. Wf or Ds were incubated with HSA (100 μM) at a ratio of 2:1 for at least 1 h at room temperature before complex **1** was added at final concentrations of 200, 400, or 800 μM. The final concentration of methanol was 10% in all samples. Calibration curves for Wf or Ds in the range of 50–300 μM were determined before the start of each displacement study, to allow for accurate quantification of the amounts of Ds or Wf displaced by complex **1**.

### Capillary electrophoresis

All measurements were performed in at least triplicate on a CE system G7100 (Agilent, Waldbronn, Germany) equipped with a long-life deuterium lamp (8 pin) with an radio-frequency identification (RFID) tag. A capillary (fused silica, 50 μm ID, Polymicro Technologies) with a total length of 50 cm and an effective length of 40 cm was installed for the separation with ultraviolet (UV) detection. The capillary zone electrophoresis (CZE)–UV data and peak areas presented were recorded at 200 nm, while the internal standard signal was recorded at 254 nm in the samples containing αMEM and human serum, as other neutral species were overlapping the signal at 200 nm. For HSA binding site studies, 210 nm was selected as the evaluation wavelength.

New capillaries were conditioned with HCl (0.1 M) and H_2_O followed by NaOH (1.0 M) and H_2_O for 10 min each followed by flushing with BGE [phosphate buffer (20 mM, pH 7.4)] for 20 min. The same procedure was carried out as daily conditioning. The capillary was flushed with BGE (3 min) before each run and was rinsed with HCl (0.1 M; 30 s), H_2_O (30 s), NaOH (1.0 M, 2 min), and H_2_O (2 min) after each separation. The sample tray and the capillary were kept at 25°C. Samples were injected hydrodynamically (50 mbar for 5 s) and the voltage applied was +20 kV for 5 min. The current reading was 23–24 μA.

For the binding site studies with HSA, a modified version of a CE method was applied.^21^ Before each run, the capillary was flushed with BGE (2 min), then +28 kV voltage was applied for 30 s followed by 1 min of flushing with BGE and the capillary was rinsed with HCl (0.1 M; 2 min), H_2_O (2 min), NaOH (1.0 M, 2 min), and H_2_O (2 min) after each separation. The sample tray and the capillary were kept at 25°C. Samples were injected hydrodynamically (30 mbar for 5 s) and the voltage applied was +28 kV for 5 min. The current reading was 34 μA.

### Method validation

Calibration curves for complexes **1**–**3** were recorded for concentrations ranging from 50 to 300 μM. The limit of detection (LOD) was defined as three times the random error in the *y*-direction (S*_y_*_/_*_x_*) of the calibration curve divided by the slope of the calibration curve.^28^ The limit of quantification (LOQ) was defined as 10 times the random error in the *y*-direction (S*_y_*_/_*_x_*) of the calibration curve divided by the slope of the calibration curve.^28^ Precision and accuracy for analysis of complexes **1**–**3** were determined at three different validation levels (100, 200, and 300 μM).

### Fourier transform–ion cyclotron resonance–mass spectrometry (FT–ICR–MS)

The adduct formation between complex **1** and Tf was further studied using a Bruker Solarix XR 7 T FT–ICR–MS. A mixture of **1** and Tf in ratio 5:1 in H_2_O (18 MΩ, Millipore) was incubated for 24 h. A sample was collected after 0 and 24 h, and the samples were frozen until analysis. The samples were diluted 1:100 with 0.1% formic acid in H_2_O (18 MΩ, Millipore) prior to analysis, producing a final concentration of 2 μM for complex **1** and 0.4 μM for Tf. A separate standard of Tf at 0.4 μM was also prepared and analyzed.

## Results and discussion

The Ru complexes [Ru^II^(cym)(8-HQ)Cl] **1**, [Ru^II^(cym)(8-HQ)(PTA)](SO_3_CF_3_) **2**, and [Ru^II^(cym)(PCA)Cl]Cl **3** are highly cytotoxic against human cancer cells.^8–10^  ^1^H NMR stability studies have shown that complex **1** and a close derivative of **3** undergo halido/aqua ligand exchange quickly in aqueous environment, while complex **2** is inert.^8–10^ Complex **3** may form subsequently dimeric structures with the S donor acting as a bridging ligand. In these hydrolyzed forms, the complexes are stable in aqueous environment for at least 3 days. However, there is limited knowledge about their interactions with biological molecules and in particular their behavior in biological environment. CZE has been used before to simulate biological conditions and investigate the stability of the compounds in an environment that resembles physiological conditions, i.e. in phosphate buffer at pH 7.4.^3^^,^^21^^,^^23^ During the CZE method development, the pre- and post-conditioning procedures were optimized to produce stable migration times over long sequences while keeping the overall run time short. We found that a sequence of washing steps with HCl, H_2_O, NaOH, and again H_2_O ensured stable migration times when running samples with a higher protein content.

Due to the limited aqueous solubility especially of complex **1**, the compounds were dissolved in methanol and then diluted with water or BGE for stability investigations. Under the conditions used and independent of the sample being diluted with water or BGE, the only peaks detected in the electropherograms (Supplementary Fig. S1) were found for species that migrated faster than the charge-neutral Co complex used as the internal standard and as the electroosmotic flow (EOF) marker. For the charge-neutral complex **1**, this indicates that it underwent ligand exchange to form a cationic species, which is in accordance with NMR spectroscopic data that suggested immediate halido/aqua ligand exchange upon dissolution in water.^9^ Both **2** and **3** feature complex cations in their structures, and are therefore expected to migrate before the EOF. While complex **2** does not feature a labile halido ligand, complex **3** can undergo halido/aqua exchange reactions and was also found to form dimeric products with the sulfur donor acting as the bridging ligand.^8^ The same samples were analyzed several times over 24 h and the peak areas assigned to the cationic species detected remained constant over this period, suggesting that the complex cations of **2** and **3**, or initial aquation products of complexes **1** and **3** are stable for at least 24 h in water, which is in accordance with previous ^1^H NMR stability studies.^8–10^

The CZE method was validated for the samples diluted with BGE and the LOD and LOQ were determined. The method gave linear calibration curves for standards ranging from 50 to 300 μM for complexes **1**–**3** (Table 1) with the LODs and LOQs as low as 19 and 62 μM for complex **1**, respectively. The accuracy of the analysis for all complexes was found acceptable (Supplementary Table S1), with some limitations for complex **1** at higher concentrations, which are however not physiologically relevant and were not used in any of the further studies. The precision for the analyses of complexes **1** and **3** were adequate with RSD values <8% and <6%, respectively, while again at higher concentration the precision of the method for complex **2** decreased.

**Table 1:** LODs, LOQs, and correlation coefficients (*R*^2^) for complexes **1**–**3**

Complex	LOD (μM)	LOQ (μM)	*R* ^2^
**1**	19	62	0.996
**2**	76	252	0.945
**3**	42	140	0.983

**Fig. 2:**
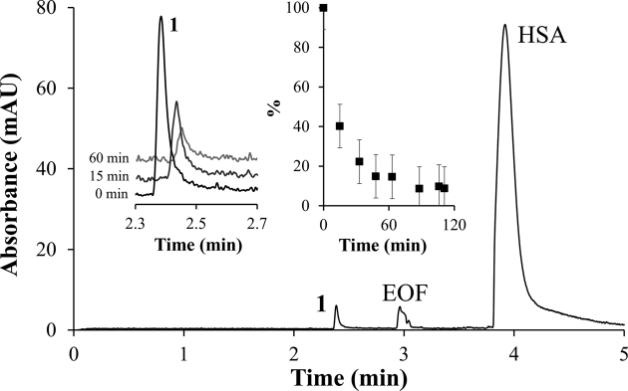
Electropherograms recorded at 200 nm in time-dependent interaction studies of complex **1** (200 μM) with HSA at a molar ratio of 10:1. The insets show the complex peak in electropherograms (left) and the peak area relative to the internal standard (right) declining over 2 h.

As the mode of action of metal-based anticancer agents often involves the interaction with proteins, the binding to the highly abundant serum proteins HSA and Tf was investigated using the same CZE method as for the stability studies. The electropherograms featured peaks for the respective protein, which are negatively charged at pH 7.4 and therefore migrate after the EOF, and a peak for the complex as observed in the stability studies (Fig. 2). For the protein-binding studies, we tracked the peak of the cationic species observed in the stability studies, as the peak area for the protein peak was considered too large relative to the change in peak area expected upon binding. Time-dependent studies over 2 h showed that complex **1** bound to HSA and Tf very quickly when incubated at a molar ratio of 1:1 (Fig. 2 and Supplementary Fig. S2). In fact, the reactions at a molar ratio of 1:1 were too quick to collect kinetic data, as the complex peak was not detectable in the first run. At a molar ratio of 10:1, the majority of complex **1** was found attached to either protein; however, the reaction reached completion significantly quicker for HSA than for Tf. Increasing the ratio to 20:1 resulted in about a quarter to a third of the complex to remain unbound after 2 h of reaction time (Supplementary Fig. S2). As the reaction with proteins proceeds very quickly at low complex:protein ratios, larger variations of the data are not surprising. In general, the rapid development of the HSA and Tf adducts is similar to the reported behavior for the Ru-based drug candidate KP1019, which, however, was found to bind quicker to Tf than HSA.^23^

**Fig. 3:**
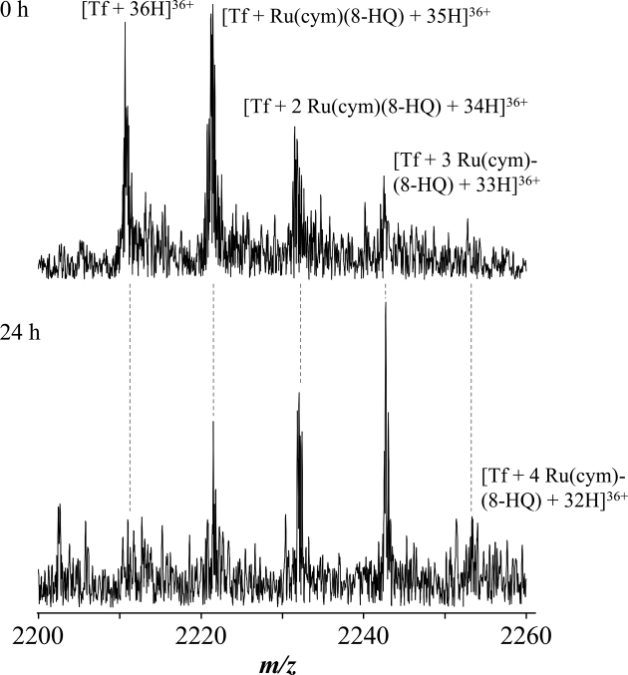
Mass spectra recorded after 0 and 24 h for a reaction mixture of [Ru^II^(cym)(8-HQ)Cl] **1** and Tf in water at a molar ratio of 5:1.

Since mass spectra for HSA usually show very broad peaks, only the adduct formation between complex **1** and Tf (molar ratio of 5:1) was further investigated with FT-ICR-MS to characterize the nature of adducts formed. FT-ICR–MS recorded immediately after mixing and after 24 h incubation suggest a quick reaction with the chlorido ligands displaced by proteinaceous amino acids (Fig. 3). The mass spectrum recorded immediately indicates adducts with one, two, and three Ru(cym)(8-HQ) moieties attached to the protein formed, i.e. [Tf + Ru(cym)(8-HQ)], [Tf + 2 Ru(cym)(8-HQ)], and [Tf + 3 Ru(cym)(8-HQ)], while unreacted protein was still detectable. The mono-adduct [Tf + Ru(cym)(8-HQ)] was the most abundant species in the mass spectrum. After 24 h, no peak assignable to free Tf was observed and the adduct species [Tf + 3 Ru(cym)(8-HQ)] was dominant, with a general shift to higher adducts.

The binding studies between [Ru^II^(cym)(8-HQ)(PTA)](SO_3_CF_3_) **2** and HSA or Tf showed that the complex does not interact with either protein. While the peak area was found to vary between measurements, these variations are most likely due to system instabilities rather than binding of the complex to proteins (Supplementary Figs. S3 and S4). Unfortunately, the use of the internal standard did not improve the data. However, overall these analyses confirm that [Ru^II^(cym)(8-HQ)(PTA)](SO_3_CF_3_) does not undergo ligand exchange for donor atoms of proteins, as was already anticipated from stability studies.

An equivalent series of studies with [Ru^II^(cym)(PCA)Cl]Cl **3** and HSA as its proteinaceous binding partner resulted in electropherograms with signals of very low intensity or peaks were entirely absent. Visual observation of the vials revealed precipitation, which was most likely the cause of the missing signals. Reducing the concentrations and changing the incubation ratios did not prevent precipitation, making any analysis of the data impossible. However, no precipitation was visible upon incubation of complex **3** with Tf (20:1 and 10:1) although the peak areas for complex **3** and in particular Tf were relatively small compared to the stability studies. The decrease in peak area of complex **3** may indicate binding to Tf and in particular the difference in peak area for incubation mixtures at 10:1 and 20:1 molar ratios compound:protein, i.e. 64% to 37% supports this hypothesis. However, the decrease in Tf peak areas for the two incubation ratios (62% versus 82%, respectively) suggests at least some protein precipitation. Therefore, the method does not allow drawing conclusions for complexes like **3** and its protein-binding ability, as it may induce precipitation of the protein.

**Fig. 4:**
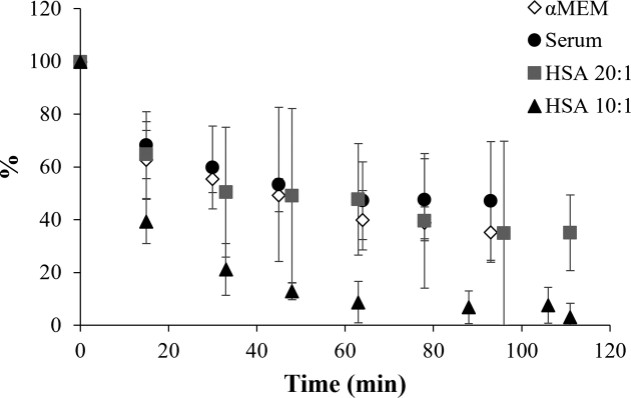
Relative peak area for [Ru^II^(cym)(8-HQ)Cl] at different times and varying complex:protein ratios (*n* = 3).

Moving from incubations with isolated proteins to more complicated matrices, we studied the interactions of the complexes with αMEM spiked with 5% FBS (diluted 1:10, corresponding to a complex:albumin ratio of 133:1) and human serum (diluted 1:100, corresponding to a complex:albumin ratio of about 50:1) to simulate the behavior after administration to the blood stream, as well as in cell biological investigations. While proteins such as albumin are a major component, this matrix also contains a variety of small molecules that may interact with the metal complex. Complex **1** after incubation with αMEM spiked with 5% FBS and human serum showed a similar behavior in terms of binding as found for the incubation with HSA at a ratio of 20:1 (complex:protein; Fig. 4). This is despite using a much higher concentration of complex **1** relative to HSA, which demonstrates that in both matrices there are other potential binders in addition to HSA. However, in neither matrix was all of complex **1** completely consumed as found for the 10:1 complex-to-HSA incubation (Fig. 4). For complex **2**, the PTA derivative of complex **1**, the binding studies with αMEM spiked with 5% FBS or serum resembled the data obtained with the isolated proteins and no interactions were observed (Supplementary Fig. S5). Similarly for complex **3**, as in the HSA binding studies, precipitation was observed shortly after adding the complex to the diluted αMEM or human serum and therefore the data could not be evaluated.

Since [Ru^II^(cym)(8-HQ)Cl] **1** was the only complex to show binding to HSA, it was chosen for further studies to identify its binding site(s) on HSA. Displacement studies with the site markers Wf and Ds for sites I or II on HSA, respectively, were carried out by CZE. For the displacement studies, Ds and HSA (100 μM) were preincubated at a ratio of 2:1, which resulted in ca. 50% of the Ds bound to HSA and still allowed detection of a peak for unbound Ds. Addition of 200, 400, or 800 μM of complex **1** displaced Ds to different degrees (Table 2). While incubation with 200 μM of [Ru^II^(cym)(8-HQ)Cl] **1** had hardly any effect on the level of bound Ds, increasing the concentration to 400 and 800 μM of complex **1** resulted in an increase of the Ds peak in the electropherograms, indicating displacement of Ds by complex **1**, with the highest concentration sample causing immediate and quantitative release of the site marker.

**Table 2:** Addition of different concentrations of [Ru^II^(cym)(8-HQ)Cl] **1** to displace Ds from HSA (*n*  = 3)^a^

	% Ds bound		
Concentration of complex 1/μM	0 h	24 h	48 h
200	44 ± 6	50 ± 7	45 ± 7
400	35 ± 7	19 ± 11	33 ± 7
800	Fully displaced	Fully displaced	Fully displaced
	% Wf bound		
	0 h	24 h	48 h
200	58 ± 4	59 ± 4	51 ± 4
400	26 ± 9	23 ± 5	33 ± 8
800	Fully displaced	Fully displaced	Fully displaced

^a^Before addition of complex **1** as the competitive binder, 50% Ds or 69% Wf were bound to HSA.

The same series of experiments was conducted with Wf. The incubation of Wf and HSA (100 μM) at a molar ratio of 2:1 resulted in 69% of the Wf being bound to HSA. The addition of complex **1** displaced Wf to different degrees, similar to the studies with Ds (Table 2). While the addition of 200 μM of complex **1** did not affect the Wf loading, 400 μM of complex **1** was not able to fully displace Wf, even after 48 h of incubation. Increasing the concentration to 800 μM of **1**, however, fully displaced Wf immediately, as was seen with Ds. These results clearly show that [Ru^II^(cym)(8-HQ)Cl] **1** was able to displace both Wf and Ds from HSA, indicating that the complex binds to both sites I and II on HSA at higher molar ratios, making HSA a potential binder after intravenous administration. The binding to HSA has been suggested to result in deactivation of drugs;^13^^,^^14^ however, this may not apply to metallodrugs like complex **1** as the compound still showed significant anticancer activity despite cell culture medium containing considerable amounts of protein. This is similar to the observations we made before for Ru drug candidates^29^^,^^30^ and others for Pt(IV) prodrugs,^31^ where the binding to HSA may contribute to the accumulation in the tumor by exploiting the enhanced permeability and retention effect.^32^

## Conclusions

A CZE method was developed, validated, and applied to study the stability and biomolecule interactions of the anticancer organoruthenium complexes [Ru^II^(cym)(8-HQ)Cl] **1**, [Ru^II^(cym)(8-HQ)(PTA)](SO_3_CF_3_) **2**, and [Ru^II^(cym)(PCA)Cl]Cl **3**. The stability studies resembled those done by other methods and demonstrated long-term stability upon initial ligand exchange reactions occurring in aqueous media. [Ru^II^(cym)(8-HQ)Cl] **1** was found to quickly bind to the serum proteins HSA and Tf. Similarly, analysis of incubation mixtures with αMEM and human serum showed a rapid decline of the peak assigned to the complex. FT-ICR-MS investigations on the adduct formation of [Ru^II^(cym)(8-HQ)Cl] **1** with Tf indicated binding of several [Ru(cym)(8-HQ)] moieties to the protein and virtually no unbound [Ru^II^(cym)(8-HQ)Cl] **1** was detected after 24 h. Through displacement studies by CZE, it was shown that [Ru^II^(cym)(8-HQ)Cl] **1** can bind to both sites I and II on HSA. [Ru^II^(cym)(8-HQ)(PTA)](SO_3_CF_3_) **2** was designed to be inert and, unsurprisingly, did not react with any of the proteins. In contrast, the incubation of HSA or Tf with [Ru^II^(cym)(PCA)Cl]Cl **3** caused precipitation, which does not allow to draw conclusions on the protein-binding ability of this compound.

This study with a series of organometallic compounds using a newly developed and validated CE method verifies its applicability as a quick way to investigate the stability and biomolecule interactions of potential anticancer agents under conditions simulating the biological environment. The difference in behavior observed between the complexes shows how important the choice of ligand is in the design of novel compounds and allows the control of interactions with serum proteins, which will have a major impact on the mode of action.

## Supplementary Material

mfac043_Supplemental_Files

## Data Availability

The data underlying this article are available in the article and in its online supplementary material.
