# Supplementary material for: The aqueous stability and interactions of organoruthenium compounds with serum proteins, cell culture medium, and human serum
Source: Metallomics. 2022 Jul 25;14(7):mfac043. doi: 10.1093/mtomcs/mfac043 (PMC9314723; doi:10.1093/mtomcs/mfac043)
Supplement: mfac043_Supplemental_Files [file mfac043_supplemental_files.zip › Supporting_Information_Revised.docx]

**Supporting Information**

**to**

**The aqueous stability and interactions of organoruthenium compounds with serum proteins, cell culture medium and human serum**

Mie Riisom,^a^ Liam Eade,^a^ William D. J. Tremlett,^a^ Christian G. Hartinger^a,^*

*^a^ School of Chemical Sciences, University of Auckland,* *23 Symonds Street, Auckland 1010, New Zealand*

**Table of Contents**

Electropherograms for stability studies

Precision and accuracy data

Stability and protein binding data

**
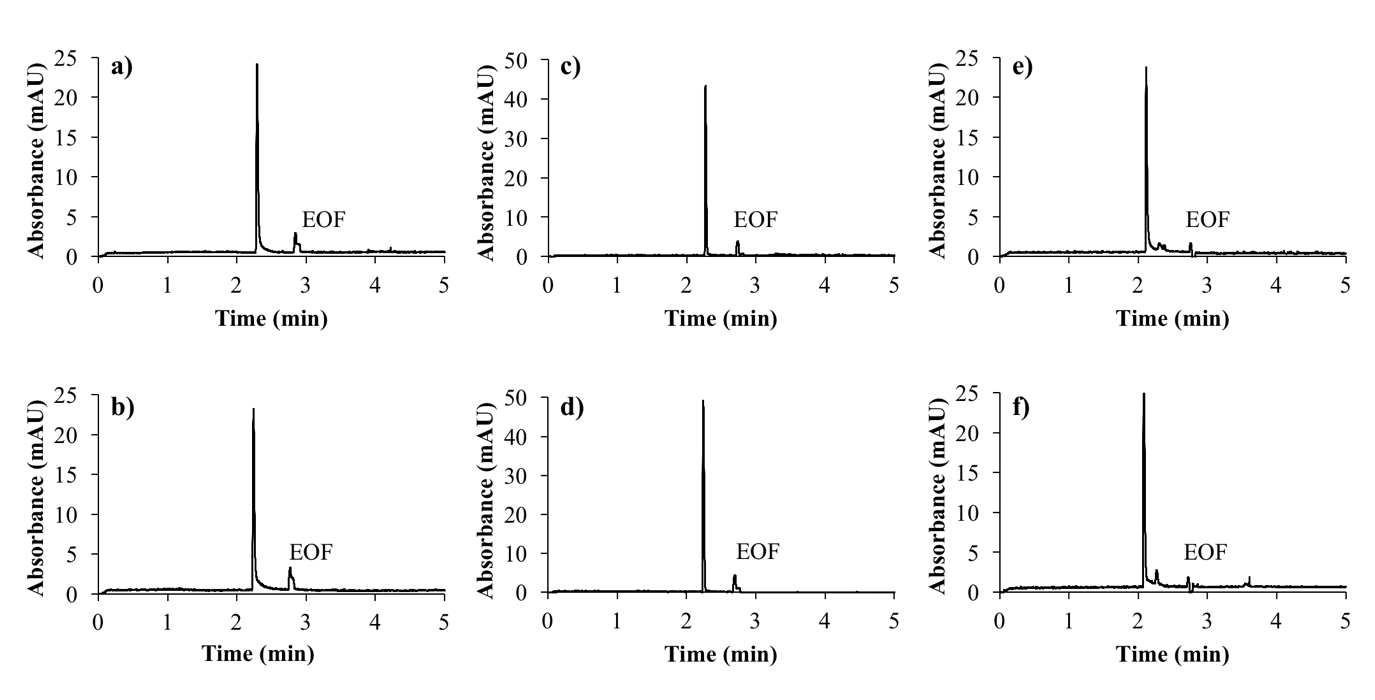
**

**Figure S1.** Electropherograms recorded at 200 nm during stability studies of **1**–**3** (200 μM) in methanol/BGE after 0 and 24 h incubation periods. a) **1**, 0 h; b) **1**, 24 h; c) **2**, 0 h; d) **2**, 24 h; e) **3**, 0 h; f) **3**, 24 h.

**Table S1.** Precision and accuracy for determination of **1–3** (n=5).

| **Complex** | **Validation parameter** | **Theoretical concentrations** | | |
| --- | --- | --- | --- | --- |
|  |  | 100 µM | 200 µM | 300 µM |
| **1** | % Accuracy ± SD | 125 ± 9 | 117 ± 11 | 154 ± 28 |
|  | % RSD | 7.4 | 4.5 | 6.1 |
| **2** | % Accuracy ± SD | 101 ± 11 | 116 ± 20 | 115 ± 52 |
|  | % RSD | 10 | 8.7 | 15 |
| **3** | % Accuracy ± SD | 102 ± 2 | 93 ± 8 | 113 ± 19 |
|  | % RSD | 2.1 | 4.6 | 5.6 |


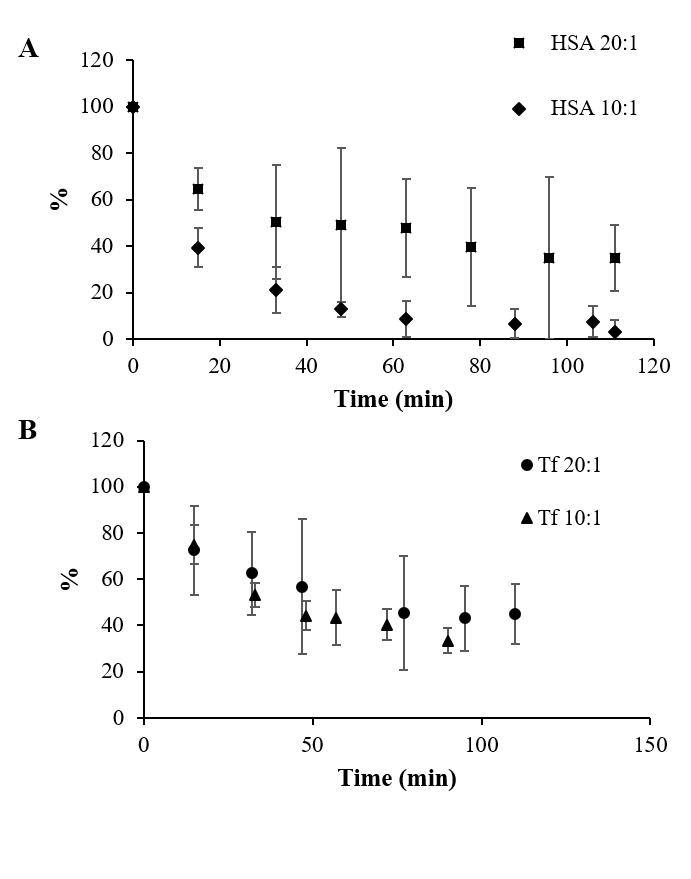


**Figure S2.** Peak areas for [Ru^II^(cym)(8-HQ)Cl] **1** relative to the internal standard [Co(acac)_3_] at different times and varying complex : protein (**A**: HSA; **B**: Tf) ratios (n = 3), recorded at 200 nm.


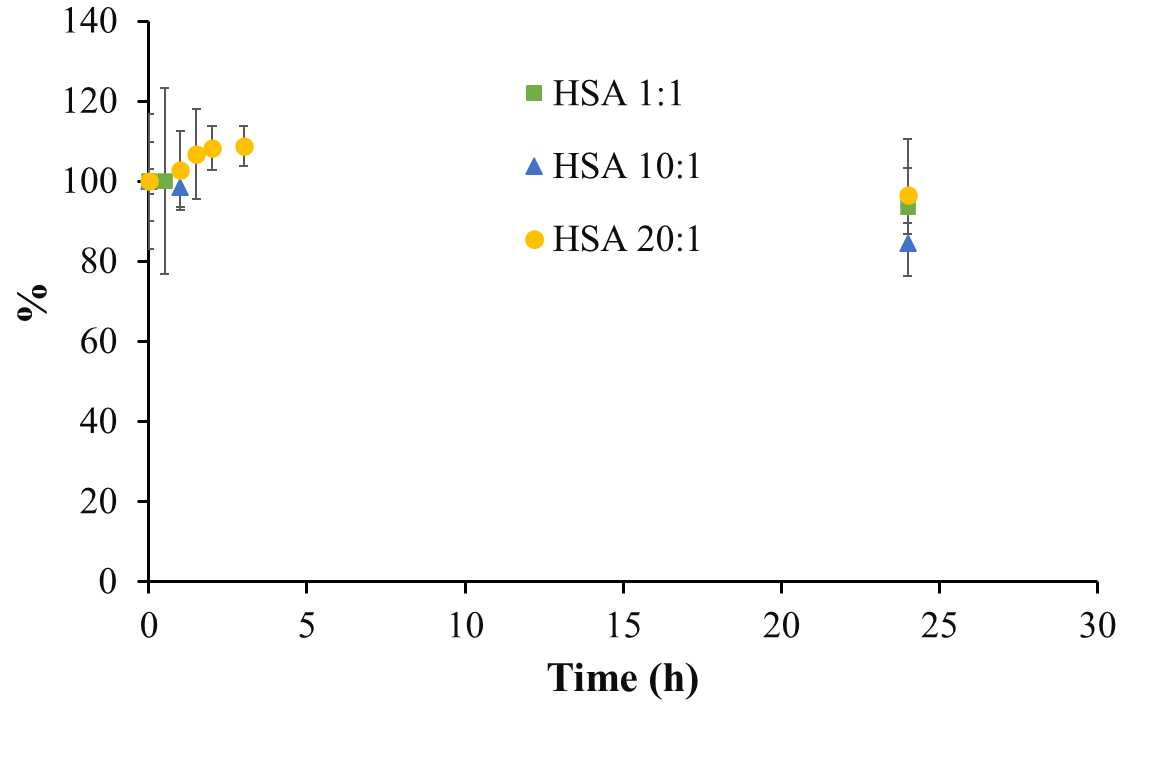


**Figure S3.** Relative internal standard-corrected peak area changes for the interaction of **2** with HSA at varying complex : protein ratios over 1 d (n = 3), recorded at 200 nm.

**
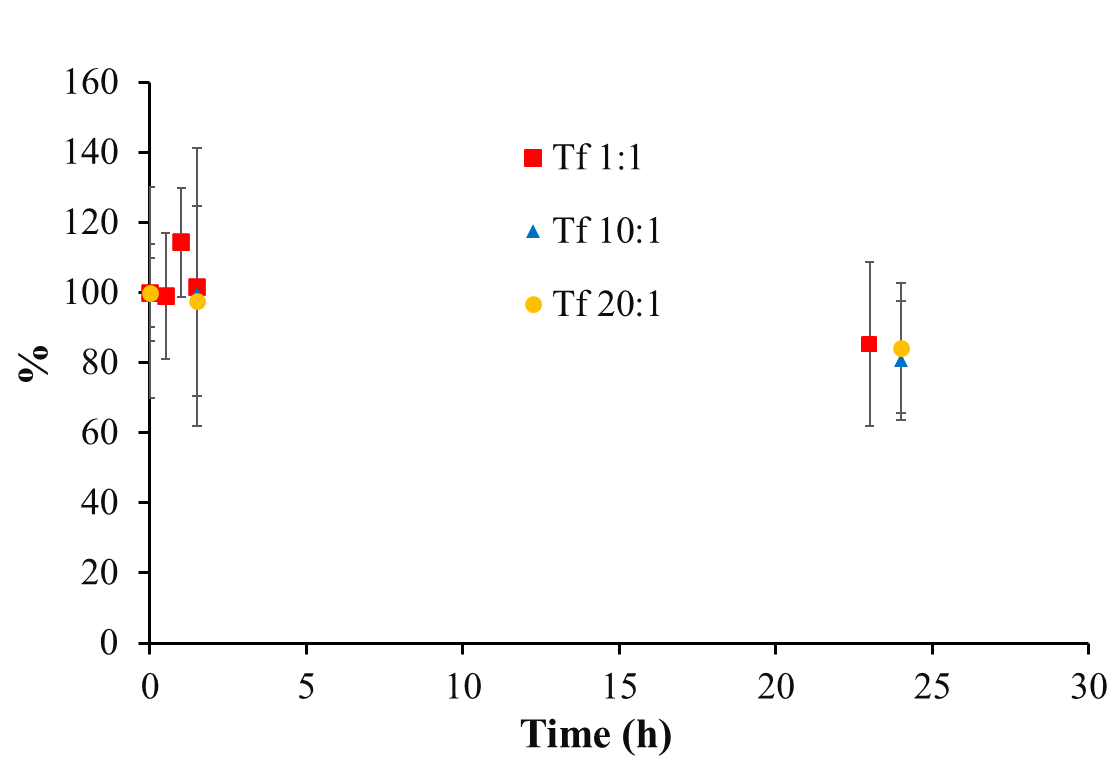
**

**Figure S4.** Relative internal standard-corrected peak area changes for the interaction of **2** with Tf at varying complex : protein ratios over 1 d (n = 3), recorded at 200 nm.


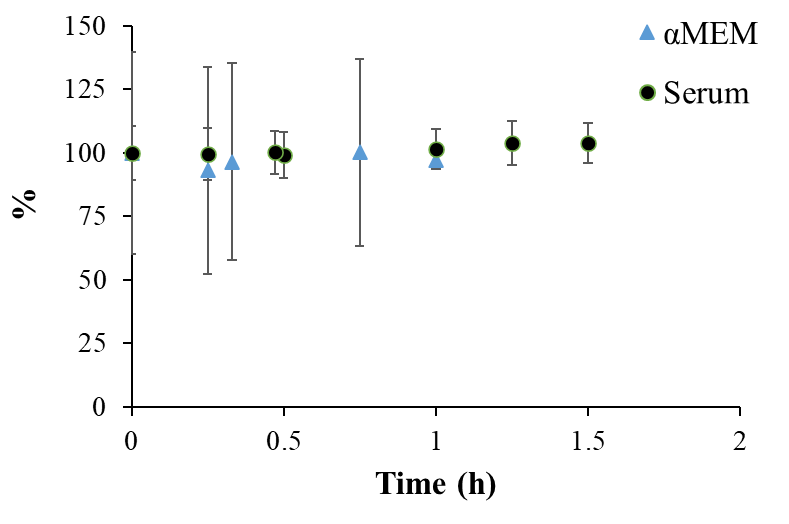


**Figure S5.** Relative internal standard-corrected peak area changes for the interaction of **2** with αMEM or serum over 1.5 h (n = 3), recorded at 200 nm for the complex and at 254 nm for the internal standard.
