# Supplementary figures and images for: The aqueous stability and interactions of organoruthenium compounds with serum proteins, cell culture medium, and human serum
Source: Metallomics. 2022 Jul 25;14(7):mfac043. doi: 10.1093/mtomcs/mfac043 (PMC9314723; doi:10.1093/mtomcs/mfac043)

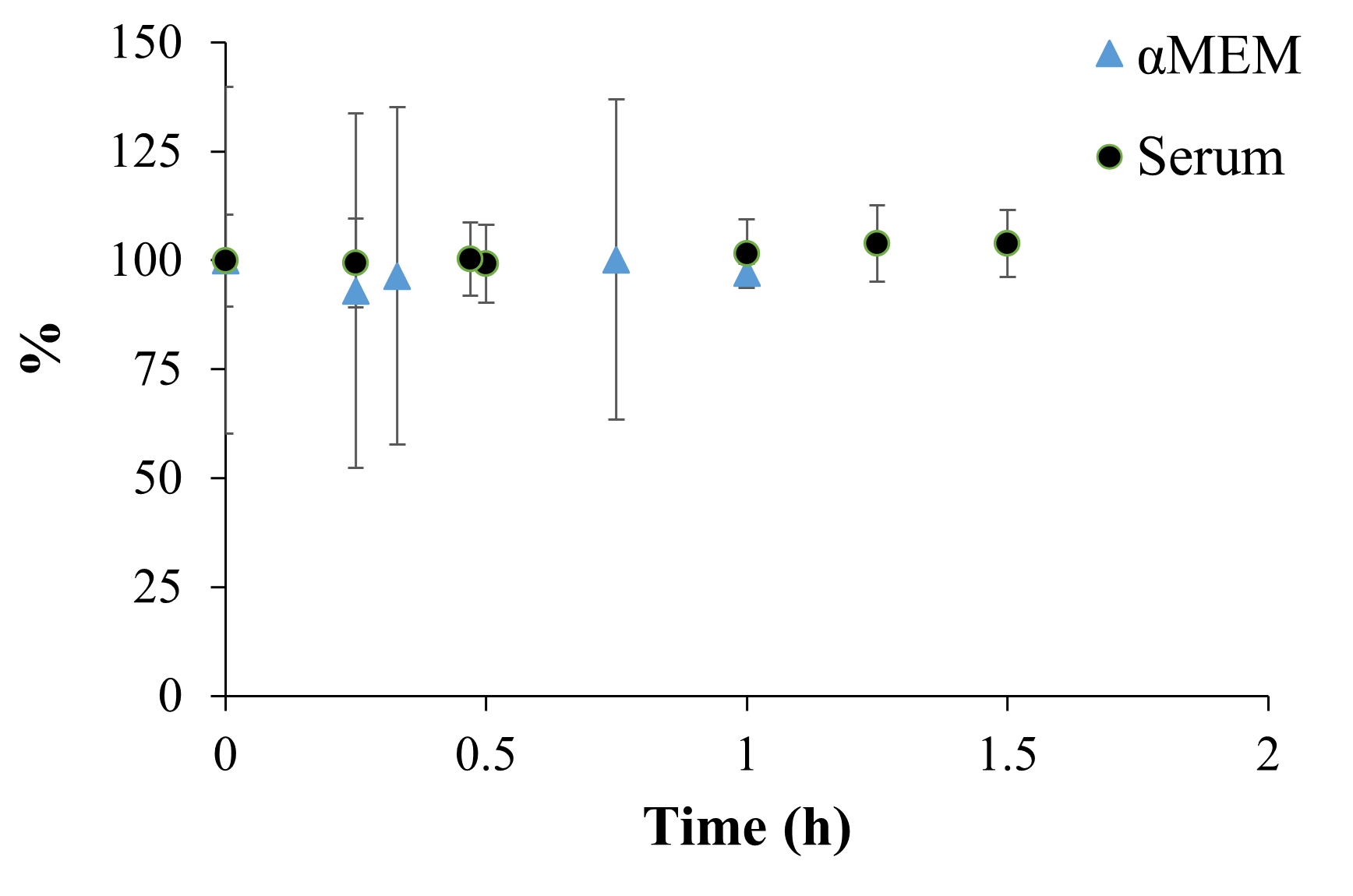

Supplement: mfac043_Supplemental_Files [file mfac043_supplemental_files.zip › suppl_data-Figure_S5.tif]

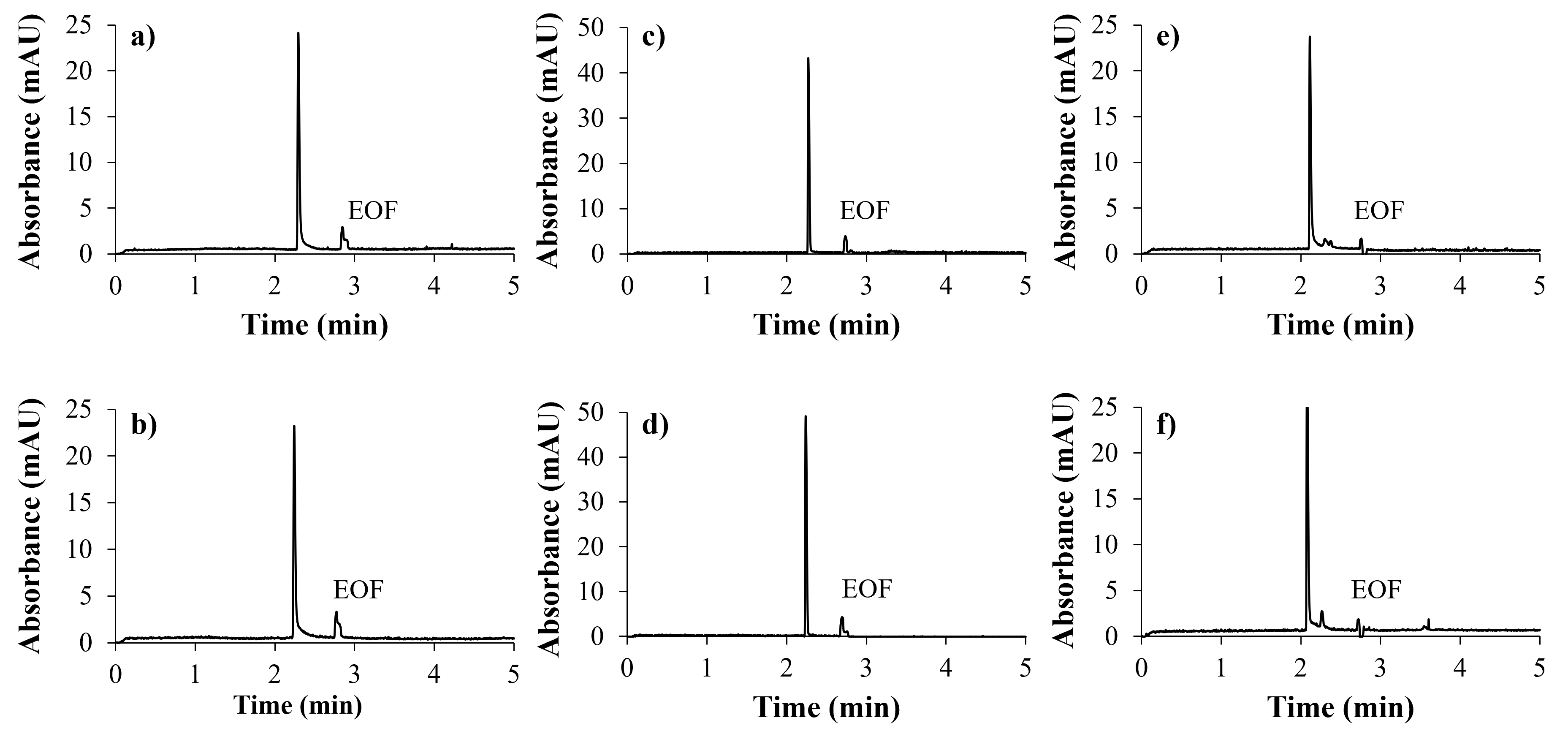

Supplement: mfac043_Supplemental_Files [file mfac043_supplemental_files.zip › suppl_data_Figure_S1.tif]

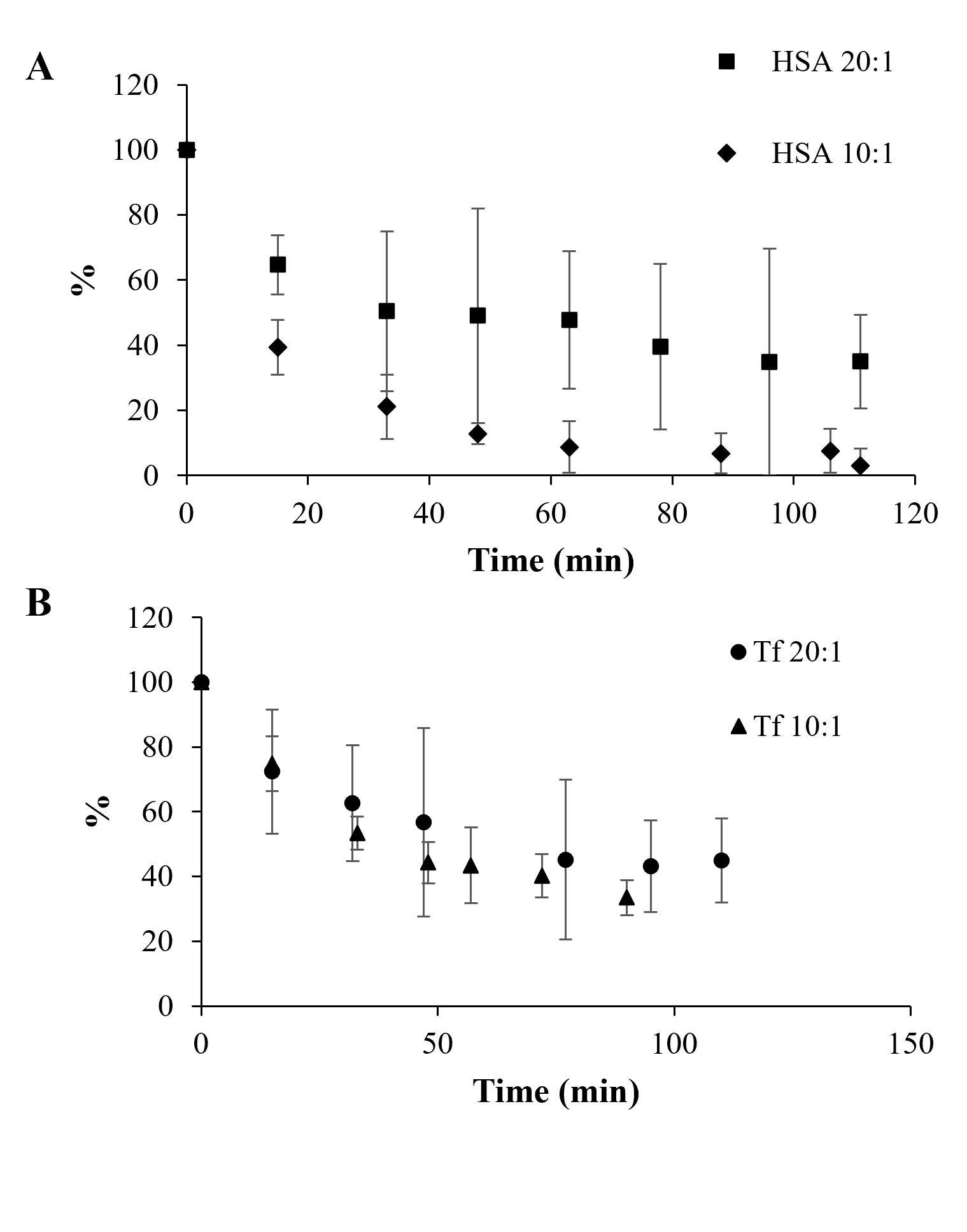

Supplement: mfac043_Supplemental_Files [file mfac043_supplemental_files.zip › suppl_data_Figure_S2.tif]

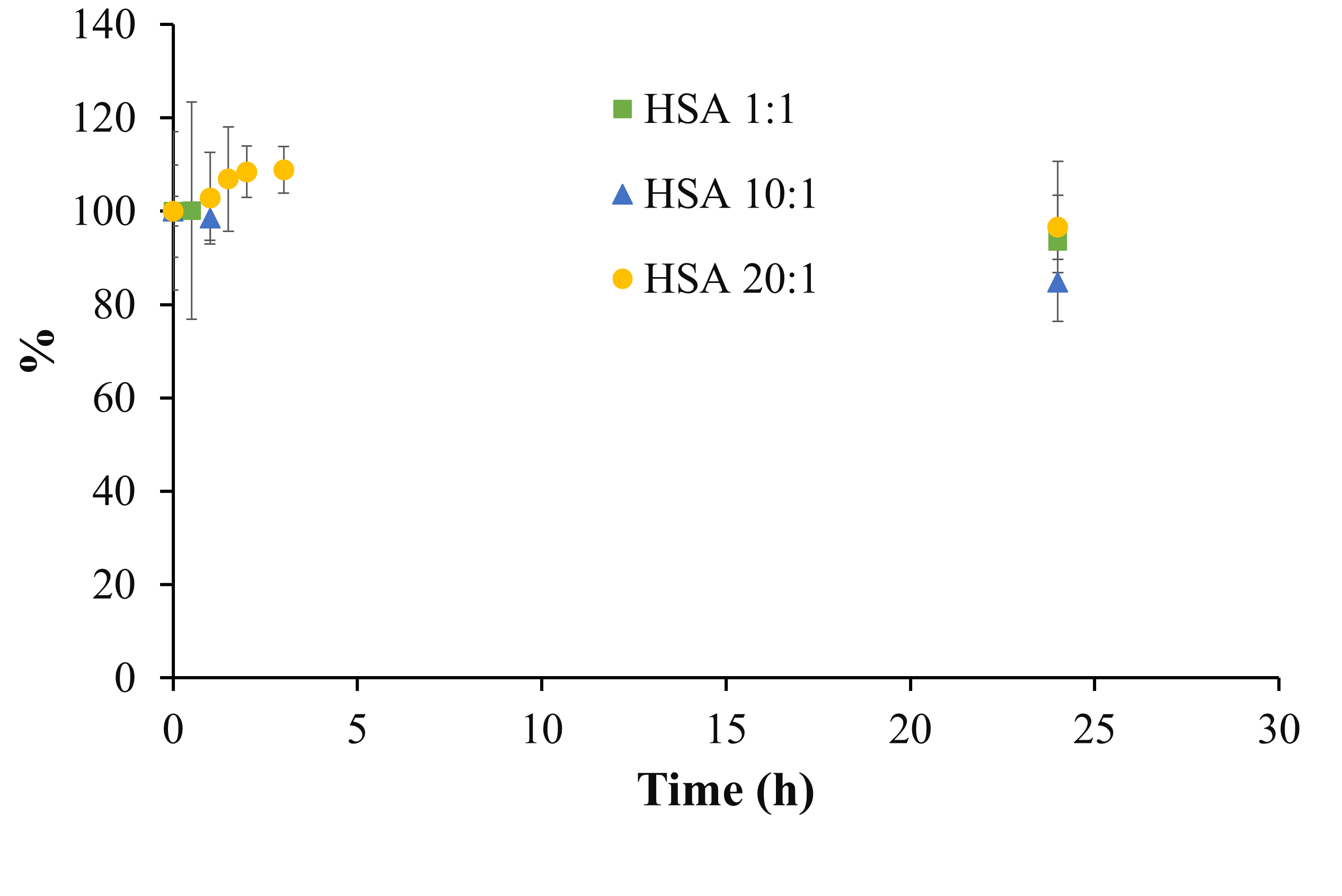

Supplement: mfac043_Supplemental_Files [file mfac043_supplemental_files.zip › suppl_data_Figure_S3.tif]

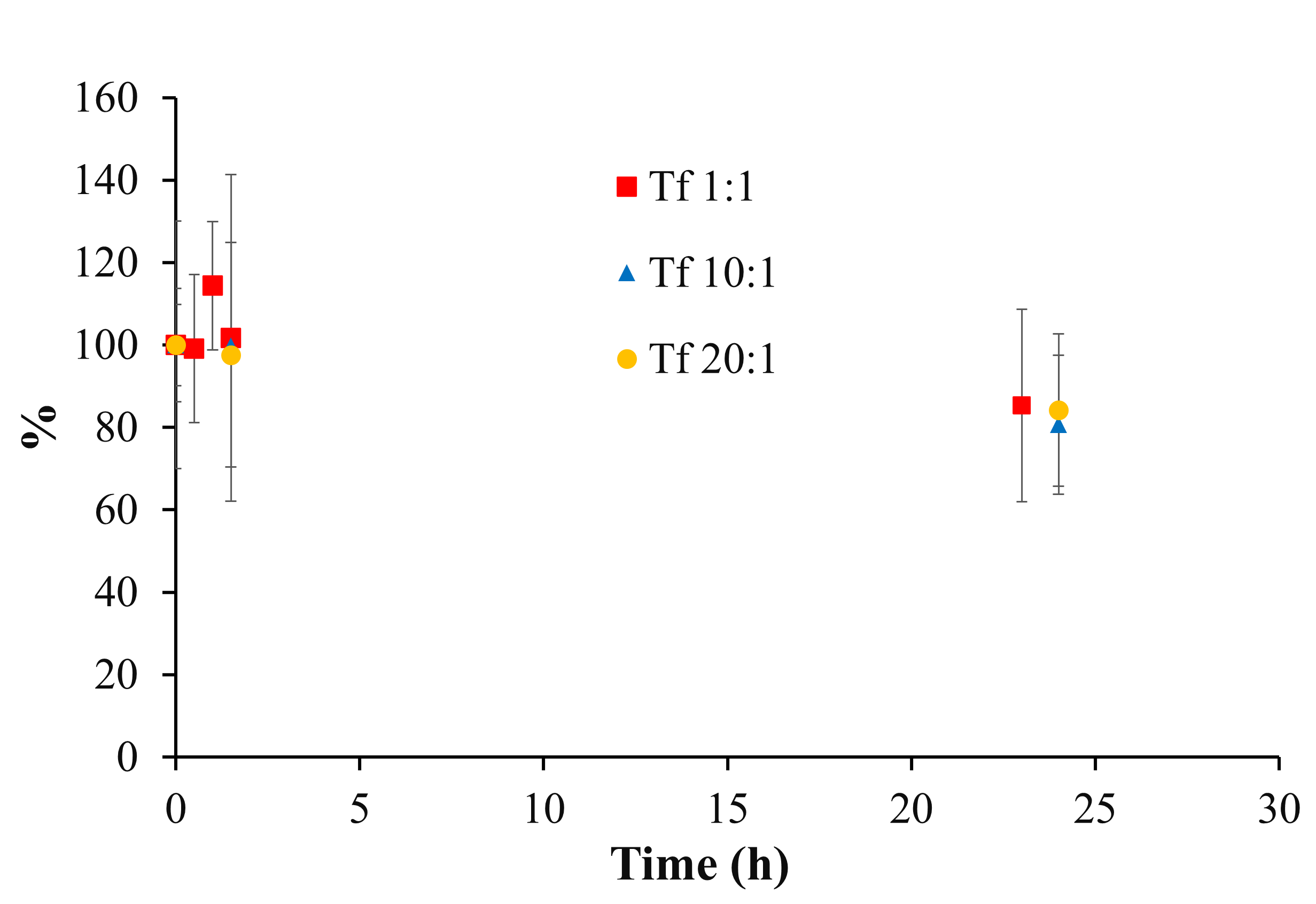

Supplement: mfac043_Supplemental_Files [file mfac043_supplemental_files.zip › suppl_data_Figure_S4.tif]
